# Lung adenocarcinoma expressing receptor for advanced glycation end-products with primary systemic AL amyloidosis: a case report and literature review

**DOI:** 10.1186/s12885-016-3009-3

**Published:** 2017-01-05

**Authors:** Shouichi Okamoto, Shinsaku Togo, Ichiro Nagata, Kazue Shimizu, Yoshika Koinuma, Yukiko Namba, Jun Ito, Toshimasa Uekusa, Kazuhisa Takahashi

**Affiliations:** 1 Department of Respiratory Medicine, Juntendo University School of Medicine & Graduate School of Medicine, 2-1-1 Hongo, Bunkyo-ku, Tokyo, 113-8421 Japan; 2 Research Institute for Diseases of Old Ages, Juntendo University Graduate School of Medicine, Tokyo, Japan; 3 Junior Resident of Juntendo University Hospital, Tokyo, Japan; 4 Department of Pathology, Labor Health and Welfare Organization Kanto Rosai Hospital, Kanagawa, Japan

**Keywords:** Amyloidosis, Case report, Lung adenocarcinoma, RAGE

## Abstract

**Background:**

Receptor for advanced glycation end-products (RAGE), a receptor for amyloids, is constitutively expressed in lungs and generally observed to be downregulated in lung cancer tissues. However, increasing levels of RAGE or serum amyloids is associated with poor outcome in lung cancer patients. We report a rare case of primary systemic amyloid light-chain (AL) amyloidosis in biopsy-proven multiple organs with early-stage non-small cell lung cancer (NSCLC) that displayed strong staining for RAGE in the tumour tissue. Interestingly, compared with randomly selected lung cancer biopsy samples, including all representative histological subtypes of NSCLC and small-cell lung cancer, only the NSCLC in the present case showed strong expression for RAGE that can bind amyloids.

**Case presentation:**

A 71-year-old woman was admitted to our hospital for comprehensive investigation of nephrotic syndrome. Computed tomography showed a small nodule in the right upper lung lobe with hilar mediastinal lymph node enlargement. Pathological examination of transbronchial biopsy samples of the nodule yielded a diagnosis of lung adenocarcinoma. Furthermore, the pathological detection of amyloid deposition in biopsy samples of a subcarinal lymph node, gastric and duodenal mucosa, cardiac muscle, and bone marrow led to a diagnosis of primary systemic AL amyloidosis with nephrotic syndrome and cardiomyopathy. In addition, RAGE was detected in lung tumour tissues surrounded by normal lung tissues with amyloid deposition.

**Conclusion:**

The RAGE positivity of the lung cancer cells in this case suggests an interaction between amyloid-containing tissues and RAGE-expressing cancer cells. Lung adenocarcinoma with RAGE expression may be a complication of underlying amyloidosis.

## Background

The incidence of systemic amyloidosis in patients with cancer is very rare and has been estimated to be between 0.1% and 0.4% among all cancers [1]. Increasing serum amyloid A (SAA) level in a patient with non-small cell lung cancer (NSCLC) is considered a predictive biomarker of poor prognosis [2]. Receptor for advanced glycation end-products (RAGE) is a transmembrane receptor of the immunoglobulin superfamily and binds structurally diverse molecules, including amyloids. RAGE is constitutively expressed in lungs and observed to be downregulated in lung cancer patients. RAGE associates with survival and metastatic spread of cancers [3, 4]. Herein, we report on a rare case of primary systemic amyloid light-chain (AL) amyloidosis in biopsy-proven multiple organs with early-stage NSCLC that displayed strong staining for RAGE in the tumour tissue.

## Case presentation

A 71-year-old Japanese woman, non-smoker, with a history of cholelithiasis, hypertension, and dyslipidaemia, was referred to our hospital for evaluation of nephrotic syndrome. The patient had been diagnosed with hypertrophic cardiomyopathy 6 months previously.

**Fig. 1:**
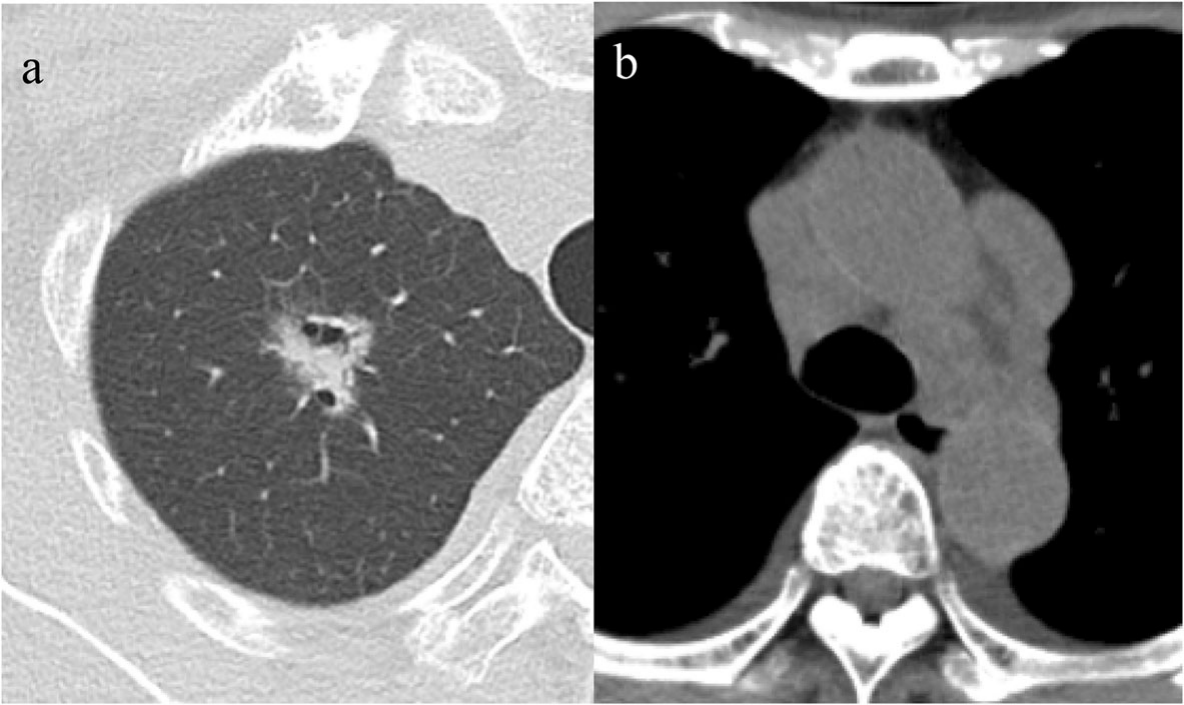
Computed tomography (CT) images. **a** Chest CT scan showing the nodule with marginal irregularity and bronchodilatation in the right upper lobe. **b** CT scan in the mediastinal window showing mediastinal lymph node enlargement and bilateral pleural effusion

On physical examination, the patient was 155.0 cm tall. She weighed 46.0 kg and showed a systolic ejection murmur from the left sternal border to the apex and pitting leg oedema. The remainder of the examination was unremarkable. On blood analysis, hypoalbuminemia (1.7 g/dL), proteinuria (4.5 g/gCr), and serum IgG M-protein were detected. Serum free light chain (SFLC) assay showed an increase in free lambda chain with a decreased kappa/lambda ratio (kappa SFLC: 7.8 mg/L, normal 3.3–19.4 mg/L; lambda SFLC: 70.5 mg/L, normal 5.7–26.3 mg/L; kappa/lambda ratio: 0.11, normal 0.3–1.3). On the other hand, SAA (5.6 μg/mL) and immunoglobulin were within normal limits. Creatinine (0.8 mg/dL), brain natriuretic peptide (325.8 pg/mL), and carcinoembryonic antigen (6.4 ng/mL) were elevated. Chest radiography showed a nodule, 2.1 cm in diameter, in the right upper lung field. Computed tomography revealed a nodule with marginal irregularity and bronchodilatation in the right upper lobe, hilar mediastinal lymph node enlargement, slight bilateral pleural effusion, pericardial effusion, and ascites (Fig. 1).

**Fig. 2:**
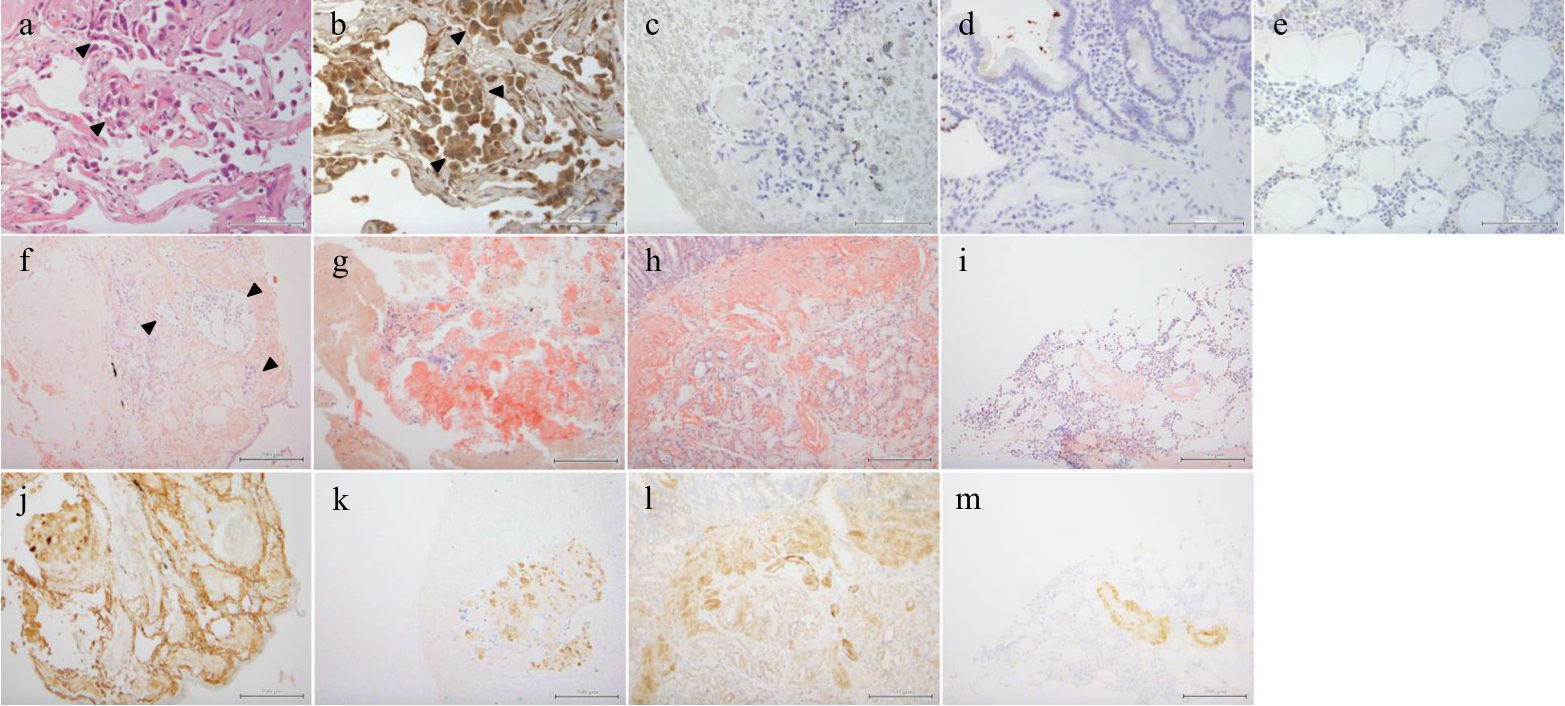
Microphotographs of the present case. **a-b** The lung adenocarcinoma (**a, b**: arrowheads): **a** haematoxylin and eosin staining (bar = 100 μm); **b** receptor for advanced glycation end-products (RAGE) staining (bar = 100 μm). Positive staining for RAGE is seen. **c-e** RAGE staining of other tissues (bar = 100 μm): **c** subcarinal lymph node; **d** duodenal mucosa; **e** bone marrow. None of these tissues show positive staining for RAGE. **f-i** Congo red staining (bar = 200 μm): **f** lung tissue surrounding the adenocarcinoma; **g** subcarinal lymph node; **h** duodenal mucosa; **i** bone marrow. Amorphous deposition was found in the tissues surrounding the tumour (**f**: arrowheads). **j-m** Amyloid P component staining (bar = 200 μm): **j** lung tissue surrounding the adenocarcinoma; **k** subcarinal lymph node; **l** duodenal mucosa; **m** bone marrow. All amorphous material shows positive staining for Congo red with apple-green birefringence in the polarized view, and amyloid P component

Pathological examination of transbronchial biopsy samples of the lung nodule yielded a diagnosis of adenocarcinoma (Fig. 2a). In addition, interstitial deposition of amorphous material that stained positively for Congo red with apple-green birefringence in the polarized view, and an amyloid P component was found in the tissues surrounding the tumour and in the subcarinal lymph node (Fig. 2f, j: lung; g, k: subcarinal lymph node). Enlargement of the subcarinal lymph node was not due to cancer metastasis. In addition, biopsy samples of gastric and duodenal mucosa, bone marrow, and cardiac muscle stained positively for Congo red with apple-green birefringence in the polarized view, and amyloid P component (Fig. 2h, l: duodenal mucosa; i, m: bone marrow). Only the subcarinal lymph node and the cardiac muscle stained positively for anti-lambda light chain antibodies. Furthermore, positive staining for RAGE was detected only in the lung tumour cells (Fig. 2b-e). The bone marrow demonstrated a normal population of plasma cells with slight atypia.

Finally, the case was diagnosed as lung adenocarcinoma, Stage IA (cT1bN0M0), and primary systemic AL amyloidosis with nephrotic syndrome and cardiomyopathy.

Because the patient displayed rapidly worsening edema and cardiac amyloidosis with elevated brain natriuretic peptide, she was given a poor prognosis rather than that expected with early-stage lung adenocarcinoma and was treated with dexamethasone (20 mg/day) and diuretics. The oedema, mainly due to the nephrotic syndrome with severe proteinuria, pleural effusion, and brain natriuretic peptide levels were not responsive to treatment. The patient died after 3 months despite dexamethasone and bortezomib treatment in another hospital.

## Discussion

RAGE is a multiligand receptor that binds structurally diverse molecules, including high mobility group box 1, S100 family of proteins, some species of advanced glycation end-products, and β-sheet fibrillar material (e.g., amyloid-β and SAA). RAGE is constitutively expressed at high levels in alveolar-type cells and at relatively low levels in vascular endothelial cells, inflammatory cells, and neurons [4–6]. RAGE and its ligands are highly upregulated in cancer tissue (e.g., pancreatic, colon, and prostate cancer) [7]. By contrast, both RAGE and serum soluble RAGE (sRAGE) levels are downregulated in smokers and lung cancer patients [7–9].

**Fig. 3:**
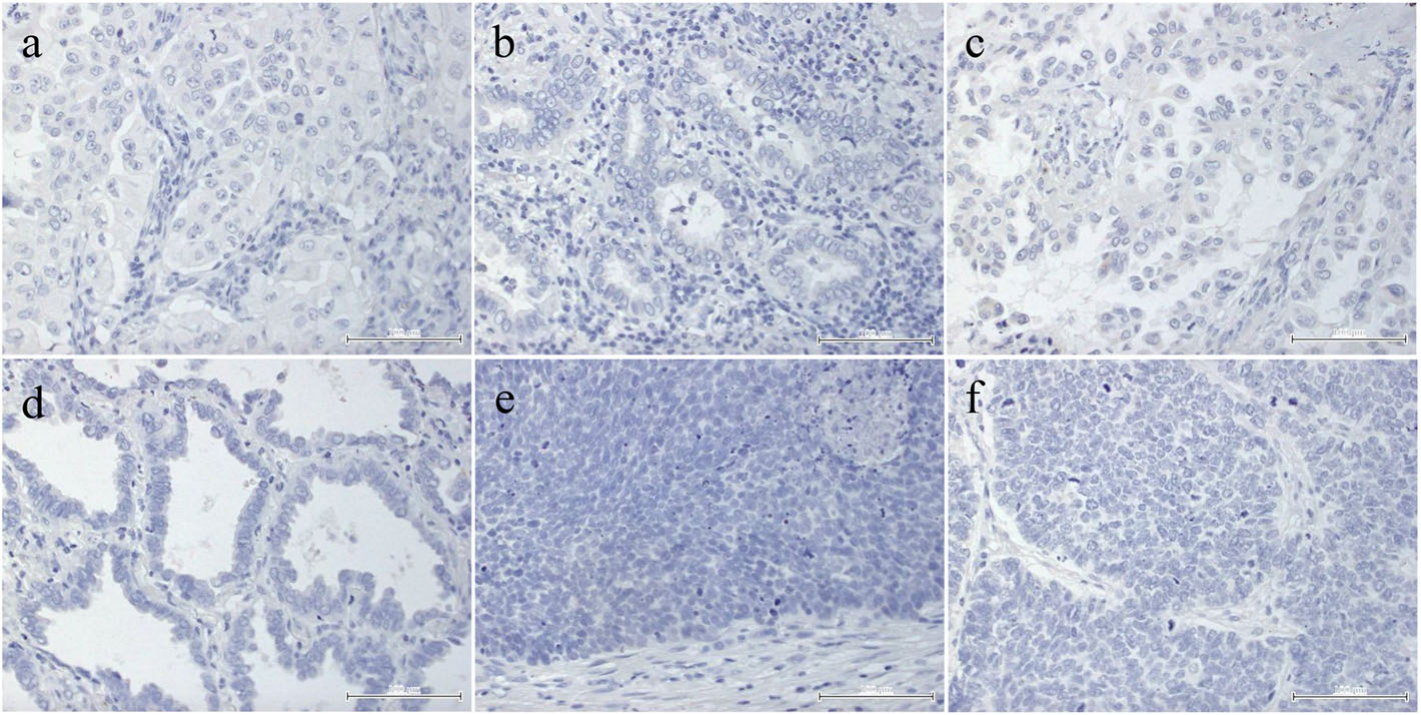
Immunohistochemical staining for receptor for advanced glycation end-products (RAGE) in control lung cancers: **a** solid adenocarcinoma; **b** acinar adenocarcinoma; **c** papillary adenocarcinoma; **d** lepidic adenocarcinoma; **e** squamous cell carcinoma; **f** small-cell carcinoma. All controls stained negatively for RAGE (bar = 100 μm)

Interestingly, RAGE that can bind amyloids showed strong expression in primary lung adenocarcinoma tissue in the early stages (Fig. 2b) and negative expression in other amyloid-positive tissues without metastasis such as the subcarinal lymph node, duodenal mucosa, and bone marrow (Fig. 2c, d, e). We confirmed RAGE staining in lung cancer tissues without comorbidity of amyloidosis by applying immunohistochemical analysis in randomly selected biopsy samples of lung cancer, including all representative histological subtypes of NSCLC and small-cell lung cancer; these samples were used as the negative control (Fig. 3a-f).

Previous studies have reported that expression levels of RAGE and its ligands are associated with clinical outcome in patients with NSCLC. Upregulated RAGE expression and activity are associated with tumour invasion and metastatic activity in certain types of neoplasia, including gastric and colon cancer [10, 11]. In contrast, overexpressed RAGE in lung cancer cells suppresses tumour growth and the acquisition of cancer stem cell features in vitro [12]. sRAGE traps circulating ligands that are overexpressed in lung cancer and thus acts as an inhibitor of RAGE-mediated cell signalling [13]. We were not able to monitor the clinical time course of serum sRAGE level as a surrogate marker in this case. There is speculation that downregulation of both RAGE and sRAGE may be a critical step in the formation of lung tumours [8, 9, 14]. Several genetic single nucleotide polymorphism (SNP) studies identified that the SNPs in the RAGE associated with increased NSCLC risk and a lower chemotherapy response rate and poor prognosis [9, 15, 16]. In addition, increasing concentrations of SAA corresponded to poor response to tyrosine kinase inhibitors and correlated with poor clinical outcome [2, 17]. In the present patient, the onset of lung cancer appeared to match mostly with that of systemic AL amyloidosis with increasing SFLC, regarded as the precursor form of amyloid protein [18]. This case was not AA amyloidosis but systemic AL amyloidosis; thus, SAA levels were within normal limits.

The relationship between cancer and amyloidosis is still unknown, as well as the relevance of amyloidosis as a paraneoplastic syndrome induced by lung cancers. However, 10 of 12 case reports, including the present case, showed the diagnosed period of lung cancer to be the same as or prior to that of amyloidosis (Table 1) [19–29]. These clinical time courses suggest the prior onset of lung cancer may contribute to the deposition of amyloid through paraneoplastic mechanisms. In the present case, the deterioration of cardiac amyloidosis directly led to death, as a poorer prognostic factor than early lung cancer itself. Generally, RAGE levels are downregulated in lung cancer patients. However, our case showed strong expression of RAGE that was surrounded by lung tissue with amyloid deposition, even though the patient had early-stage lung cancer (Fig. 2b, f).

**Table 1:** Clinicopathological features of lung cancer patients with amyloidosis

Author, year	Age(y)/sex	Cancer type and stage	Amyloid organ involvement	Type	Prior diagnosis^a^	Treatment for lung cancer	Cause of death
van Bronswijk et al. 1982 [19]	71/M	SCLC, Advanced	K	AA	Lung cancer	Radiotherapy	Pulmonary infection
Meyrier et al. 1985 [20]	59/M	SCC, TbN1Mx	A, K, SP	AA	Lung cancer	CCRT	Renal failure due to amyloidosis
Focan et al. 1985 [21]	70/M	SCC, Early	BM, Digestive tract	N/A	Unknown	None	Pulmonary infection
Richmond et al. 1990 [22]	72/M	SCC, Early	Multiple organs	AA	Same	None	Peritonitis and Renal failure due to amyloidosis
Benharroch et al. 1992 [23]	51/F	AC, N/A	F	AL	Unknown	None	Renal failure due to systemic sclerosis
Partidge et al. 2000 [24]	69/M	NSCLC, Stage IV	Mediastinal LN	N/A	Same	None	NSCLC
Garthwhite et al. 2003 [25]	64/M	SCC, N/A	K, L	AA	Same	Radiotherapy	N/A
Barcelo et al. 2003 [26]	33/M	SCC, Stage IIIB	C, K	AA	Lung cancer	Chemotherapy	Pulmonary infection
Paydas et al. 2005 [27]	50/M	AC, Stage IIIB	K	AA	Lung cancer	CCRT	N/A
Miyazaki et al. 2011 [28]	60/M	AC, Stage IA	K, L, ST	AL	Same	Surgery	N/A
Gueutin et al. 2013 [29]	56/M	AC, Stage IIIB	K	AA	Lung cancer	Chemotherapy	N/A
Our case 2016	71/F	AC, Stage IA	D, H, K, L, ST	AL	Same	None	Heart failure due to amyloidosis

*A* adrenal gland, *AA* amyloid A amyloidosis, *AC* adenocarcinoma, *AL* amyloid light-chain amyloidosis, *BM* bone marrow, *C* colon, *CCRT* concurrent chemoradiotherapy, *D* duodenum, *F* female, *H* heart, *K* kidney, *L* lung, *LN* lymph node, *M* male, *N/A* not available, *NSCLC* non-small cell lung carcinoma, *SCC* squamous cell carcinoma, *SCLC* small-cell lung cancer, *SP* spleen, *ST* stomach, *y* years

^a^ ‘Same’ refers to a diagnosis given at the same time as amyloidosis

Thus, the RAGE positivity of lung cancer cells in this case suggests an interaction between amyloid-containing tissues and RAGE-expressing cancer cells, which may progress both lung cancer and amyloidosis. Further study is warranted to investigate this association.

## Conclusion

We describe a rare case of amyloid receptor-positive lung adenocarcinoma with systemic AL amyloidosis. Clinicians should be aware that RAGE-positive lung cancer may be a complication of underlying amyloidosis that could impact more severely on the prognosis of the patient than the cancer itself.

## Abbreviations

AL: Amyloid light-chain
NSCLC: Non-small cell lung cancer
RAGE: Receptor for advanced glycation end-products
SAA: Serum amyloid A
SFLC: Serum free light chain
SNP: Single nucleotide polymorphism
sRAGE: soluble receptor for advanced glycation end-products
